# HOXA11 gene is hypermethylation and aberrant expression in gastric cancer

**DOI:** 10.1186/s12935-014-0079-7

**Published:** 2014-08-19

**Authors:** Yinguo Bai, Na Fang, Tingxun Gu, Yuhua Kang, Jiang Wu, Desheng Yang, Hui Zhang, Zhimin Suo, Shaoping Ji

**Affiliations:** 1 Department of Gastroenterology, Huaihe Hospital of Henan University, Kaifeng 475000, Henan Province, China; 2 Department of Biochemistry and Molecular Biology, Medical School of Henan University, Kaifeng 475004, Henan Province, China; 3 Department of pathology, Huaihe Hospital of Henan University, Kaifeng 475000, Henan Province, China

**Keywords:** Gastric cancer, HOXA11 gene, Methylation, Expression

## Abstract

**Background:**

Aberrant DNA methylation is an acquired epigenetic alteration that serves as an alternative to genetic defects in the inactivation of tumor suppressor genes and other genes in diverse human cancers. Gastric carcinoma is one of the tumors with a high frequency of aberrant methylation in promoter region. Hence we investigated the promoter methylation status and expression level of HOXA11 gene which may involve in GC development.

**Methods:**

Thirty-two surgical excised gastric cancer specimens, twelve paired adjacent non-cancerous specimens and seven normal gastric mucosas were examined. The methylation status and expression level of HOXA11 gene were determined by bisulfite sequencing polymerase chain reaction (BSP), real-time polymerase chain reaction (RT-PCR) and immunohistochemistry (IHC) respectively. HOXA11 expression was knocked-down with siRNA to mimic HOXA11 gene hypermethylation and ability of cell proliferation and migration was determinate. In addition, we analyzed and correlated the findings with clinicopathological features.

**Results:**

The methylation level of HOXA11 gene in gastric cancer tissues and adjacent non-cancerous tissues were higher than those in normal gastric mucosa (P < 0.05). The methylation level was higher in TNM III and IV patients of GC than those in TNM I and II patients (P < 0.05). The expression of HOXA11 mRNA and protein decreased in normal gastric mucosa, peri-cancer tissue and GC (P < 0.05). HOXA11 expression was inversely correlated with DNA methylation (P < 0.05). Knocked-down of HOXA11 expression with siRNA in BGC-823 cells enhanced cell proliferation compared with control, but no significant different was observed in migration ability.

**Conclusion:**

Hypermethylation and decreased expression of HOXA11 gene may be involved in the carcinogenesis and development of GC and may provide useful information for the prediction of the malignant behaviors of GC. And the expression of HOXA11 is impaired by DNA methylation. However, repression of HOXA11 expression promoted BGC-823 cell proliferation.

## Background

Gastric cancer (GC) is one of the most common malignancies of the digestive system and is a major cause of cancer death in China. Because gastric cancer lacks early detective and effective curing methods, especially to intermediate or advanced stage ones, that have a poorer survival. Understanding the molecular mechanism that characterize cell growth, cell cycle, apoptosis, angiogenesis and invasion has enabled us to use new approaches to treat this disease in clinical practice. Gastric carcinogenesis is thought to be a multistep process that involves multiple genes, and epigenetic alterations in oncogenes, tumor suppressor genes, DNA repair genes, cell cycle regulators and signaling molecules, which play an important role in the occurrence and progression of GC. DNA methylation is one of the important research aspects in epigenetics [1],[2]. Gene silencing associated with aberrant methylation of CpG islands is an acquired epigenetic alteration that serves as an alternative to genetic defects in the inactivation of tumor suppressor and other genes in human cancers. A number of genes have been found to be aberrantly methylated in gastric cancer [3]-[6].

As specialized transcription factors, HOX genes play crucial roles in modulating embryonic morphogenesis and cell differentiation of the mammal, and are closely correlated to tumorigenesis [7],[8]. HOX genes in mammals are arranged into clusters (A, B, C, and D) on four different chromosomes. The HOXA cluster, located within a 155-kb-long genomic region on chromosome 7p15-7p14.2 consists of 12 genes (11 HOX genes and EVX1). Highly dense CpG islands are prevalent in most of the HOXA promoters and the hypermethylation of these islands plays pivotal roles in the control of HOXA gene expression. Among HOXA genes, HOXA11 hypermethylation has recently been reported in endometrial carcinoma, ovarian cancer, glioblastoma multiforme and cervical cancer [9]-[12]. Nonetheless, epigenetic changes and the effect of HOXA11 on gastric cancer remain unclear. Thus, relationship between HOXA11 hypermethylation and tumor development currently becomes an active exploring area in recent years.

In this study, we sought to identify potential targets of methylation induced gene silencing, in which gene expression is down-regulated in response to aberrant methylation. We have analyzed the methylation status of HOXA11 promoter and evaluated its correlation with gene expression level, as well as with different clinicopathological parameters. SiRNA was used to knock-down HOXA11 expression and mimic hypermethylation of HOXA11. Effects of repression of HOXA11 expression on cell proliferation and migration were determinate in BGC-823 cells, in which repression of HOXA11 expression augments the cell proliferation compared with control.

## Results and discussion

### Results

#### DNA methylation profile of HOXA11 gene in gastric cancer tissues

**Figure 1:**
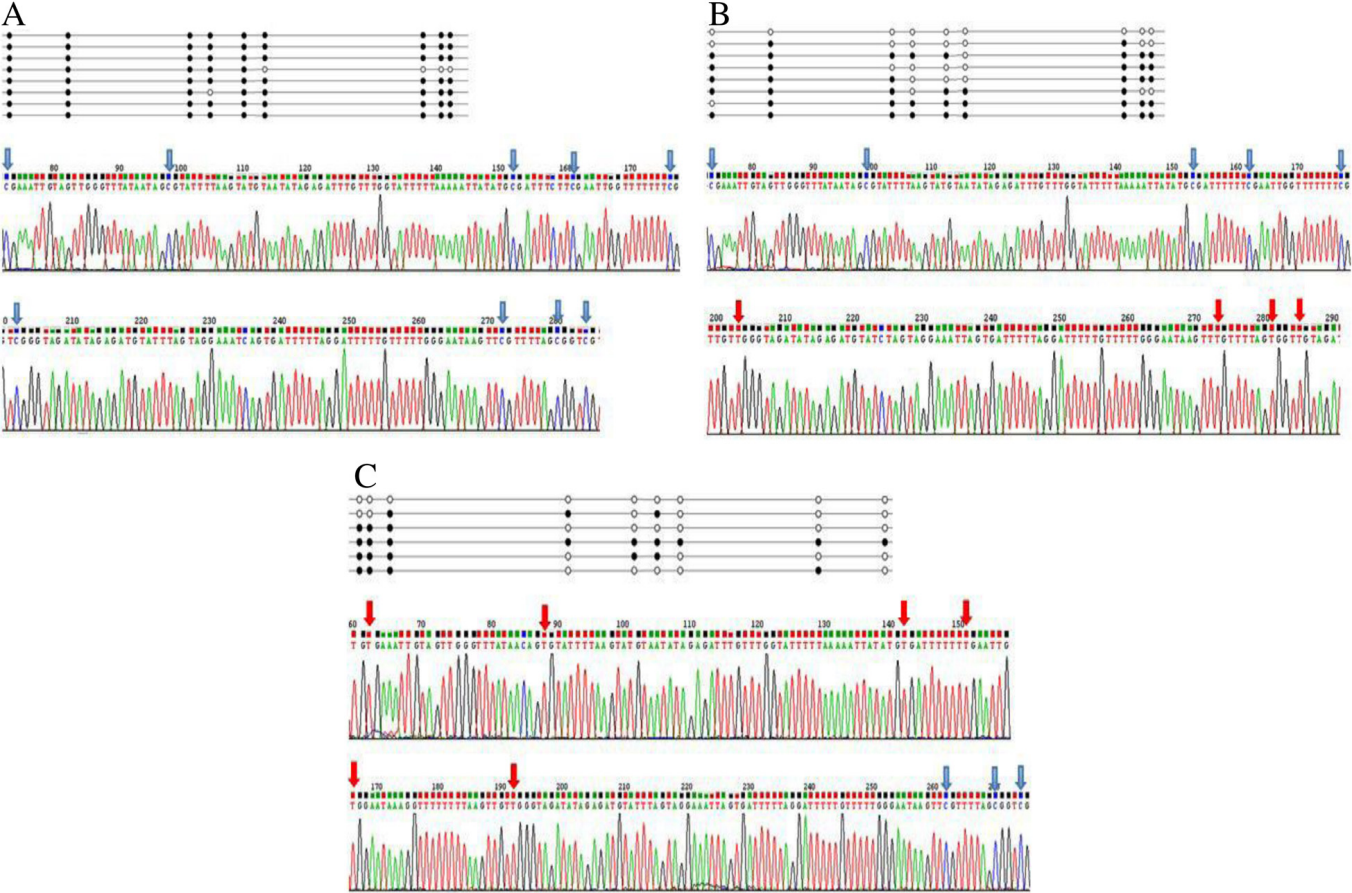
**Bisulfate-sequencing of HOXA11.** Each row represents an individual cloned allele. Circles represent CpG sites and their spacing accurately reflects the CpG density of the region. The black circles and blue arrows show methylated CpG sites; the white circles and red arrows show unmethylated CpG sites. There are two representations of graph under the each row. **(A)** gastric cancer tissue, **(B)** peri-cancer tissue, **(C)** normal gastric mucosa.

The methylation frequencies of HOXA11 gene in gastric cancer tissues, peri-cancer tissue and normal gastric mucosa were 65.028±27.4645, 61.325±24.4066, 33.886±25.6900. The methylation frequencies of HOXA11 in cancer tissues, peri-cancer tissue specimens were higher than those in normal gastric mucosa (*P =* 0.007, *P =* 0.035), but no difference was seen between the adjacent non-cancerous specimens and normal gastric mucosa (*P <* 0.05). Representative results of bisulfite sequence analysis for gene promoters in gastric cancer samples are shown in Figure 1.

#### Clinico-pathological correlations with methylation profile

The methylation level of HOXA11 gene was higher in TNM III and IV patients of GC than that in TNM I and IIpatients (*P <* 0.05, Table 1). Promoter methylation of HOXA11 was found to be associated with lymph node metastasis (*P <* 0.05, Table 1). No significant association was found between HOXA11 methylation status and other clinicopathological features such as age, gender, tumor size, differentiation degree and invasive depth in GC (*P*> 0.05, Table 1).

**Table 1:** Clinico-pathological correlations with methylation profile

**Variable**	**HOXA11**	**P value**
Age at surgery (years)		0.191
≥60	23(61.648±29.7620)	
<60	9(73.667±19.2414)	
Gender		0.211
Female	12(55.992±34.7000)	
Male	20(70.450±21.2387)	
Tumor size (cm)		0.237
≥5 cm	17(70.494±28.7441)	
<5 cm	15(58.833±25.4674)	
Pathological stage		0.049
I, II	13(53.554±28.5911)	
III, IV	19(72.879±24.3713)	
Differentiation degree		0.642
Well	20(63.240±29.0453)	
Poor	12(68.008±25.5554)	
Invasive depth		0.478
Within muscle layer	10(59.810±29.2551)	
Penetrating muscle layer	12(67.400±26.9784)	
Lymph node metastasis		0.049
Positive	22(71.414±23.8900)	
Negative	10(50.980±30.7814)	

Data were analyzed by *t* test.

#### Expression level of HOXA11 mRNA in gastric cancer

**Figure 2:**
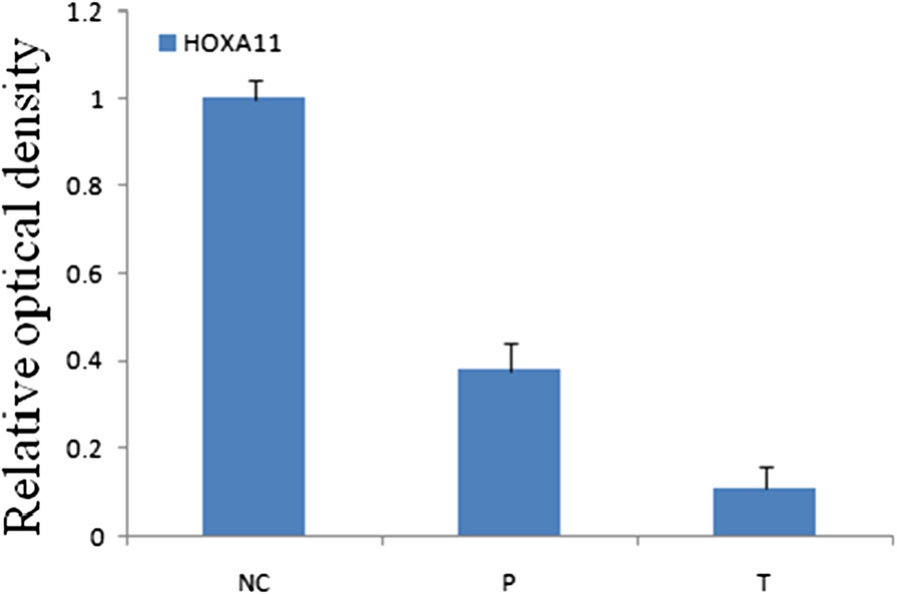
**Analysis of HOXA11 gene expression by quantitative real-time reverse transcription-polymerase chain reaction.** (NC) normal gastric mucosa, (P) peri-cancer tissue, (T) gastric cancer tissues.

The expression levels of HOXA11 mRNA were significantly lower in human gastric cancer tissues than in peri-cancer tissue and normal gastric mucosa (*P <* 0.05, Figure 2). We found a marked decrease in HOXA11 levels correlating with higher stages of tumour. This was statistically significant when comparing TNM I,II to TNM III, IV (*P <* 0.05). There was a noticeable trend with lower levels correlating with increasing age and lymphatic metastasis. However, the mRNA expression of HOXA11 gene had no relation to gender, tumor size, differentiation degree and invasive depth in GC (*P* > 0.05, Table 2).

**Table 2:** Clinico-pathological correlations with mRNA expression

**Variable**	**mRNA expression of HOXA11**	**P value**
Age at surgery (years)		0.034
≥60	22(0.4418±0.2177)	
<60	8(0.2563±0.1419)	
Gender		0.776
Female	11(0.3773±0.2217)	
Male	19(0.4011±0.2163)	
Tumor size (cm)		0.669
≥5 cm	16(0.3763±0.2246)	
<5 cm	14(0.4107±0.2099)	
Pathological stage		0.039
I, II	13(0.4838±0.1889)	
III, IV	17(0.3224±0.2118)	
Differentiation degree		0.336
Well	19(0.4216±0.1827)	
Poor	11(0.3418±0.2632)	
Invasive depth		0.103
Within muscle layer	10(0.4830±0.0733)	
Penetrating muscle layer	20(0.3470±0.1961)	
Lymph node metastasis		0.018
Positive	20(0.3285±0.2068)	
Negative	10(0.5200±0.1766)	

Data were analyzed by *t* test.

#### Analysis of immunohistochemistry

**Figure 3:**
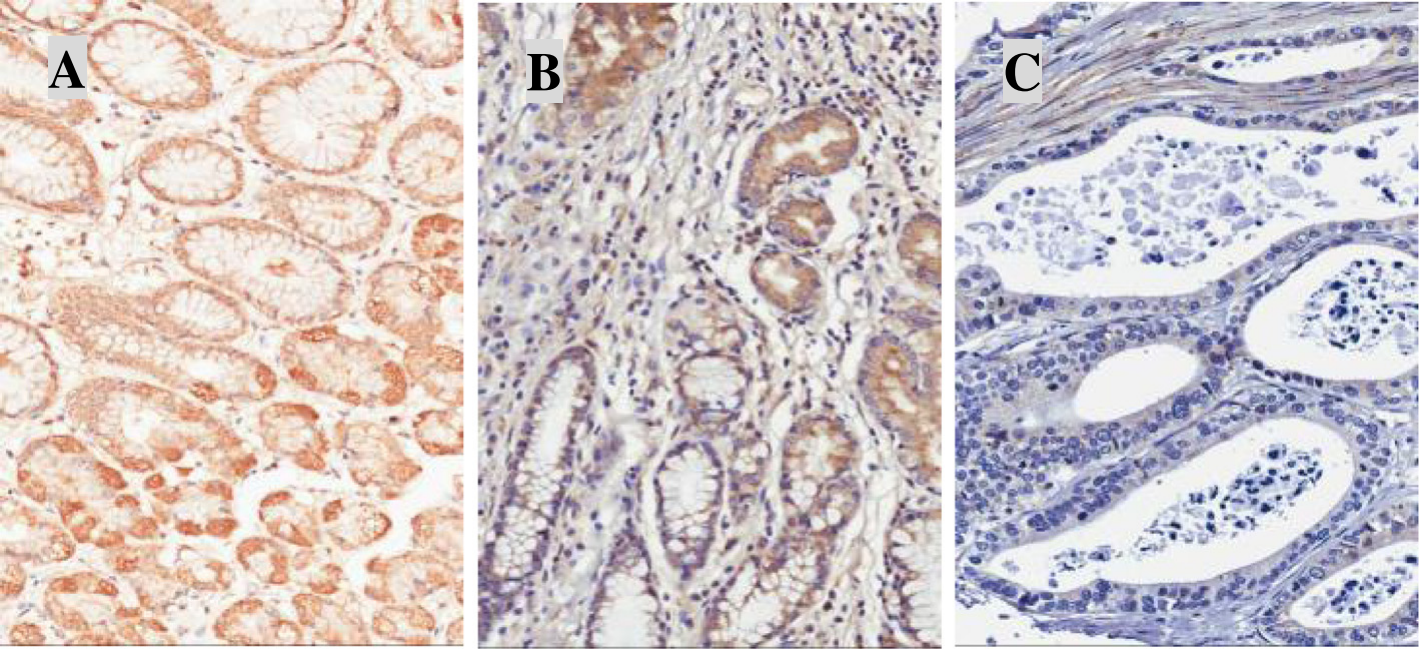
**Immunohistochemistry (IHC) of HOXA11 protein in gastric mucosa/cancer tissues which have different methylation levels of HOXA11 gene promoter regions. (A)** A typical staining of normal gastric mucosa tissues. **(B)** A typical staining of adjacent gastric mucosa tissues. **(C)** A typical staining of gastric cancer tissues. Methylation levels of HOXA11 promoter: A<B<C.

HOXA11 protein expression was then determined by IHC. Normal gastric mucosa tissue and the adjacent non-tumoral gastric mucosa had remarkably higher HOXA11 expression than GC tissues which has a highest methylation level of HOXA11 (Figure 3C). A significant decreased of HOXA11expression was observed in the adjacent non-tumoral gastric mucosa with higher methylation level than normal tissue (Figure 3B). In contrast, normal tissue has a high expression of HOXA11, but low methylation of HOXA11 promoter (Figure 3A).

#### Repression of HOXA11 expression and cell proliferation and migration

**Figure 4:**
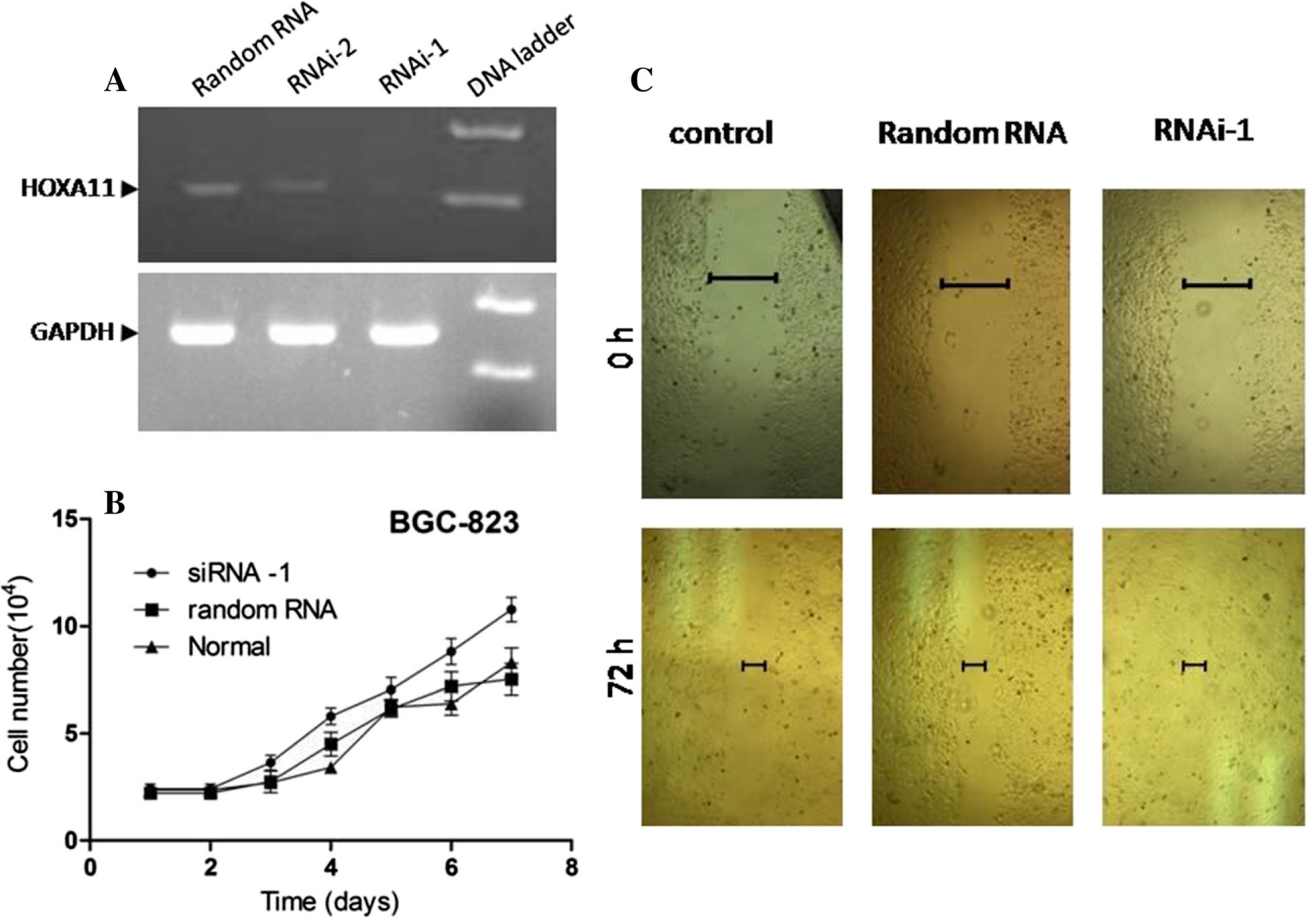
**Ability of BGC-823 cell proliferation and migration was determined when HOXA11 expression was knocked-down with siRNA. (A)** Reverse-PCR revealed fragment-1 can efficiently knock down HOXA11 expression compared with fragment-2 or random control RNA. **(B)** Cell growth curve was drawn following repression of HOXA11 expression. **(C)** Wound healing assay was determined after a reverse transfection of RNAi and random RNA. The normal cells were used as control.

Two double-stranded RNA fragments were transfected into GGC-823 cells with random RNA as control. The result of RT-PCR shown that fragment-1 more efficiently repressed HOXA11 expression compared with fragment-2 or random RNA (Figure 4A). For cell proliferation and migration assay, a reverse transfection was carried out with fragment-1 to knock-down HOXA11 expression, mimicking repression of HOXA11 expression by promoter hypermethylation. The results shown knocked-down of HOXA11 augmented the cell proliferation from third day compared with random RNA or intact cells (Figure 4B); However, in wound healing assay, we did not observe significant change of the cell migration rate when HOXA11 expression was repressed with siRNA (Figure 4C), indicating the HOXA11 defect seems to only promote cell proliferation but not augment invasion or metastasis.

### Discussion

It is currently believed that genetic and epigenetic events interact to help drive tumor progression [13]. The combination of the two sides may provide a new viewpoint to better understand the pathogenesis of gastric carcinoma [14]. Great attention has been aroused on the significance of DNA methylation for early diagnosis and prognosis prediction in malignant tumors [15]. Gastric carcinoma (GC) is one of the most frequent malignancies and remains the second leading cause of cancer-related mortality in the world [16]. Although the clinical outcome of gastric cancer has gradually improved, diagnosis of gastric carcinoma is still disappointing. Thus, early detection of gastric cancer is a key measure to reduce the mortality and improve the prognosis of gastric carcinoma [17]. Increasing evidence now suggests that, in addition to genetic alterations, epigenetic changes, including DNA methylation, histone modification and RNA interference, also play important roles in the development and progression of gastric carcinoma [18]-[20]. As a well-defined epigenetic mechanism, DNA methylation plays a major role in cancer through transcriptional silencing of critical growth regulators such as tumor suppressor genes, along with gene mutation and deletions, ultimately leading to carcinogenesis [21].

In addition to genetic causes, tumors can also be considered an epigenetic disease [22]. DNA methylation is the most important area of epigenetics which plays an important role in genomic imprinting and in the silencing of retrotransposon. In normal cells, DNA methylation may modulate compartmentalization of DNA to assure that transcriptionally active chromatin regions replicate earlier than the bulk transcriptionally inactive chromatin. Regional hypermethylation observed during tumor progression may involve in inactivating one of the two X chromosomes. These changes in chromatin structure may, through direct transcriptional inactivation of genes and allelic deletions, mediate the decreased expression of tumor suppressor genes associated with tumor development [23]. Hypermethylation of tumor suppressor genes has attracted much attention recently and DNA methylation inhibitors are being tested as potential anticancer agents [24],[25]. Drugs that inhibit DNA methylation may help patients live longer with fewer side effects than conventional cytotoxic therapy. Aberrant methylation of HOXA11 gene promoter has been found in various tumors [26]-[28], but the mechanism and roles involved in GC have not been elucidated. Therefore, further investigation into the roles of HOXA11 methylation in GC development and identification of its regulators are necessary.

Homeobox (HOX) genes were originally discovered in Drosophila melanogaster [29]. Several investigations demonstrate that HOX genes act as transcriptional regulators involved in the process of cell to cell communication during normal morphogenesis, the alteration of which may contribute to the development of cancer [30],[31]. HOX gene homology domain is able to bind to specific DNA sequences, and to regulate gene transcription [32]. However the mechanisms of HOX genes involved in tumorigenesis have not been elucidated. HOXA11 is a murine Abdominal-B-type homeobox gene that regulates lower abdominal development in Drosophila, and control differentiation of the müllerian ducts into the fallopian tubes, uterus and cervix. Studies show that low expression of HOXA11 gene has been found in various tumors, such as ovarian cancer and endometrial cancer, and it plays the role of tumor suppressor genes [33]. Previous research has shown that high methylation and low expression of HOXA11 gene is prevalent in gynecologic cancer. Because HOXA11 gene is a developmental related gene, the majority of researches have focused on its effects on the mesoderm organ development and expression. The regulation mechanism involved in tumors has not been elucidated. Cary Miller *et al*. [34] observed that Wnt7a regulates the expression of HOXA11 gene in mice, suggesting HOXA11 gene may associate with Wnt involved in regulatory network of development. In this study, we find the methylation frequencies of HOXA11 in GC tissues and adjacent cancer tissues are higher than those in normal gastric mucosa (*P <* 0.05). Moreover, expression of HOXA11 gene is down regulated when the promoter region is hypermethylated, suggesting that HOXA11 gene may play an important repressor role in GC tumorigenesis, and aberrant promoter methylation is the main reason causing loss or down-regulation of HOXA11 gene, which may be involved in the carcinogenesis of GC. Furthermore, we found that hypermethylation of HOXA11 was significantly associated with lymph node metastasis and TNM stage in gastric cancer (*P <* 0.05), but not significantly associated with other clinicopathological factors such as sex, age, tumor size, differentiation degree and invasive depth. Our results suggest that hypermethylation of HOXA11 may therefore be an important indicator of molecular biology for evaluating the degree of malignancy and lymph node status of gastric cancer. In addition, results in vitra shown HOXA11 may control cell growth and its defect enhanced the cell proliferation which may involved in carcinogenesis of GC. However, repression of HOXA11 did not significantly promote the cell migration, indicating HOXA11 defect does not augment cancer cell metastasis.

In conclusion, aberrant promoter methylation of HOXA11 is a frequent event and may be one of the main phenotypes that induces HOXA11 gene aberrant expression in GC. As a transcription regulator, loss and down-regution of HOXA11 may lead silence of cancer suppressor genes and excessive growth such as tumors. Hypermethylation of CpG islands may appear early in carcinogenesis which facilitates malignant growths, suggesting that the detection of DNA methylation from gastric juice and blood samples could serve as a molecular marker for predicting tumor progression and prognosis. DNA methylation occurred in tumors are easier to correct than to correct DNA sequence mutations or genetic damage. How to restore DNA expression by developing gene-targeting therapies by reversing aberrant methylation is currently considered a promising new approach of gastric cancer treatment. Since aberrant methylation of HOXA11 genes is significantly correlated with pathobiological behaviors in GC, analysis of DNA methylation could be used in tumor diagnosis, evaluation of chemosensitivity and prognosis.

## Conclusions

Hypermethylation of HOXA11 gene promoter regions may impair expression of HOXA11 gene. The aberrant methylation may be involved in the carcinogenesis and development of GC.

## Materials and methods

### Patients and tissue specimens

From May 2012 to March 2013, 32 patients were histologically diagnosed with gastric adenocarcinoma, and were prospectively recruited into this study from Huaihe Hospital of Henan University (Henan, China). All patients had given informed consent for specimen collection. Written informed consent was obtained from the patient for the publication of this report and any accompanying images. This study was performed with the approval of the Medical Ethical Committee of Huaihe Hospital of Henan University. The mean age of the patients (20 males and 12 females) was 63.4 years (range, 36 to 85 years, Standard Deviation 12.1), and none of the patients has received preoperative chemo- or radiotherapy. The cancerous tissue, peri-cancer tissue (located 1.5 ~ 2 cm from the primary tumor) and normal gastric mucosa (superficial gastritis patients) were bisected, one stored at –80°C immediately, the other were fixed in 4–10% buffered formaldehyde, embedded in paraffin for further use.

### DNA isolation and bisulfite sequencing (BS) analysis

Genomic DNA was extracted and bisulfite-modified using the EZ DNA Methylation-Direct Kit (Zymo Research, America) according to the manufacturer’s instructions. In brief, the tissue was chopped as fine as possible, and then added into M-Digestion Buffer^R^ with 20 mg/ml of proteinase-K. The samples were incubated at 50°C for 22 min and centrifuge at 10,000 g for 5 min. Add 20 μl of sample to 130 μl of CT Conversion Reagent^R^ solution in a PCR tube, following incubating at 98°C for 8 minutes, 64°C for 3.5 hours. Add 600 μl of M-Binding Buffer^R^ into a Zymo-Spin™ IC Column and load the sample into the Zymo-Spin™ IC Column containing the M-Binding Buffer. Mix by inverting the column several times. Centrifuge and wash samples with M-wash buffer for 2 times. The sample was eluted the in M-elution buffer.

The bisulfate modified DNA was used immediately or stored at -20°C. The transcription initiation site: -2000 bp of upstream sequence and 2200 bp of downstream sequence of the HOXA11 gene transcription initiation sites were from NCBI. CpG Island Searcher online was used for CpG islands analysis and the indicated CpG islands serve as templates for design of primers in BS. The bisulfate modified DNA was amplified by Touchdown-PCR using Takara Taq Hot Start Version (Takara, Japan). Table 3 shows the sequences of primers and annealing temperature used in the polymerase chain reaction (PCR). The PCR products were electrophoresed on 1.0% agarose gel and visualized under UV illumination. After being purified with Quick Gel Extraction Kit (CWBIO, China), PCR products were cloned into the pGEM-T vector (Promega, America). By screening of blue-white spot, ten clones of each specimen were sequenced using M13 forward or reverse primers.

**Table 3:** Primers used for RT-PCR and BSP

**Primers**	**Sense sequences 5′-3′**	**Antisense sequences 5′-3′**	**Product size (bp)**	**Annealing temperature(°C)**
HOXA11 RT-PCR	TATACCAAGTACCAGATCCGA	TGAGATCTTAATCAAGAGAGT	374	50
HOXA11 BSP	ATTTTTATATGTAAGAAATTG	AAAGTTTCCATTCTAAACAAT	276	50
β-actin	CACTGGCATCGTGATGGA	GGCCATCTCTTGCTCGAA	210	56

### Real-time reverse transcription-polymerase chain reaction quantifies HOXA11 gene expression level

Total RNA was extracted with TRIzol reagent (Invitrogen, USA) according to the manufacturer’s instructions and samples were immediately processed or stored at -80°C until use. First-strand cDNA was generated using a first strand cDNA synthesis kit (CWBIO, China). Primers used for the PCR are listed in Table 3. A qRT-PCR assay based on SYBR Green detection was carried out to examine the level of the expression of HOXA11 gene. The endpoint used in the real-time PCR quantification, Ct, is defined as the PCR cycle number that crosses an arbitrarily placed signal threshold. Gene expression was presented using a modification of the 2-ΔΔCt 2 method, first described by K. Livakin PE Biosystems Sequence Detector User Bulletin 2. The expression of each housekeeping gene was presented as 2-ΔCt, where ΔCt (ΔCtTime X-ΔCtTime 0) and time 0 represent the 1 × expression of each gene.

### Immunohistochemical staining (IHC)

The paraffin-embedded tissues blocks were sectioned at 4 μm thickness, deparaffinized in xylene, rehydrated in graded ethanol solution and endogenous peroxidase activity was blocked by incubation with 3% H_2_O_2_ in for 30 min at room temperature. Then sections were immersed in citrate-NaOH buffer (10 mM sodium citrate, pH 7.0) for 40 min at 92°C for restoration of antigenicity. The rehydrated sections were incubated overnight at 4°C with rabbit anti-human HOXA11 polyclonal antibody (1:500, ab28699, Abcam, USA). The sections incubated with the first antibody were washed with Tris-buffered saline (TBS) and then were incubated with MaxVision™ HRP-Polymer anti-Rabbit IHC Kit (Maixin, Fuzhou, China) for 15 min at room temperature. The sections were visualized using the DAB Detection Kit (Maixin, Fuzhou, China) reaction followed by counterstaining with hematoxylin. Negative control experiments were done by omitting the primary antibody. The immunohistochemical expression of HOXA11 was examined independently by two pathologists using light microscopes without information of patients. The percentage of positive tumor cells was graded semiquantitatively, and each sample was assigned to one of the following categories: 0 (0–4%); 1 (5–29%); 2 (30–59%); or 3 (60–100%). The intensity of immunostaining was determined as 0 (negative), 1 (weak), 2 (moderate), and 3 (strong). The immunoreactive score was calculated by multiplication of the grade determined by the percentage of positive cells and the staining intensity.

### Cell culture and transfection

BGC-823 cells (derived from human gastric epithelial adenocarcinoma) were cultured in DMEM (Dulbecco’s Modified Eagle Medium) with 10% bovine calf serum in 5% of CO2 and 90% of relative humidity. To knock-down HOXA11 expression, two RNA fragments were synthesized for optimizing efficiency of expression inhibition as following: Fragment-1(sense): 5′-GCCCAAUGACAUACUCCUA-3′ and fragment-2(sense): 5′-GCAGUCUCGUCCAAUUUCU-3′. After transfection and comparison by reverse-transcription PCR, fragment-1 has higher efficiency in inhibiting HOXA11 expression and was used for the following experiments. A reverse transfection was performed according to the manufacturer’s recommendations of Lipofectamine 2000 (Invitrogen) with random RNA as negative control. The cells were seeded into 24-well plates or 60-mm dishes following transfection for proliferation curve and Wound healing assay.

### Cell proliferation and migration assay

BGC-823 cells were seeded to 24-well plates (5 × 10^3^ cells/well) with reverse transfection of siRNA and cultured at 37°C with 5% CO_2_. Three duplicate wells were set up for RNAi, Random RNA and normal control. Cell number at each well was counted for each triplicate every 24 h for 7 days. For wound healing assay, cells were plated into 60-mm dishes before due day. The monolayer cells of 80% confluent were scraped with sterile 20 μL pipette tips and detached cells were washed away with warm PBS. The cells migrated into the scraped areas were photographed every day after scratch with an inverted microscopy equipped with a digital camera. Ability of wound healing among three groups was compared in parallel.

### Statistical analysis

Data were analyzed by the computer program SPSS 17.0, using analysis of *t* test. Two sided *P <* 0.05 was considered to be significant.

## Competing interests

The authors declare that they have no competing interests.

## Authors’ contributions

SJ and ZS designed the study, participated in the clone sequencing and analysis of alignment and review the final manuscript. YB and YK carried out experiments and drafted manuscript. TG completed cell counting and migration in HoxA11 knock down. NF, JW and DY helped to collect specimens and clinical information. YK and HZ analyzed data and participated in Immunohistochemistry of tissue. All authors read and approved the final manuscript.
